# Comparison of a New Test For Agility and Skill in Soccer With Other Agility Tests

**DOI:** 10.2478/v10078-012-0053-1

**Published:** 2012-07-04

**Authors:** Mehmet Kutlu, Hakan Yapıcı, Oğuzhan Yoncalık, Serkan Çelik

**Affiliations:** 1 Kırıkkale University, Education Faculty, TURKEY.; 2 School of Physical Education and Sport, Kırıkkale University, TURKEY.; 3 Kırıkkale University, Education Faculty, Department of Physical Education and Sport, TURKEY.; 4 Kırıkkale University, Education Faculty, Department of Computer Education and Instructional Technologies, TURKEY.

**Keywords:** agility, decision making, power, soccer, talent, shooting, goal

## Abstract

The purpose of this study was both to develop a novel test to measure run, shuttle run and directional change agility, and soccer shots on goal with decision making and to compare it with other agility tests. Multiple comparisons and assessments were conducted, including test-retest, Illinois, Zig-Zag, 30 m, Bosco, T-drill agility, and Wingate peak power tests. A total of 113 Turkish amateur and professional soccer players and tertiary-level students participated in the study. Test-retest and inter-tester reliability testing measures were conducted with athletes. The correlation coefficient of the new test was .88, with no significant difference (p> 0.01> 0.01) between the test results obtained in the first and second test sessions. The results of an analysis of variance revealed a significant (p < 0.01) difference between the T-drill agility and power test results for soccer players. The new agility and skill test is an acceptable and reliable test when considering test-retest reliability and inter-rater reliability. The findings in this study suggest that the novel soccer-specific agility and shooting test can be utilized in the testing and identification of soccer players’ talents.

## Introduction

Soccer is one of the most popular sports in the world, especially in Europe. Soccer is characterized by numerous short, explosive exercise bursts interspersed with brief recovery periods over an extended period of time (90 minutes) (Meckel et al., 2009). Soccer performance, which depends on the technical skills and physical fitness of the players, is known to significantly influence match performance. The simultaneous use of both technical skills and fitness in soccer training would produce extremely effective performance (Little and Williams, 2007).

Agility, acceleration, change of direction, deceleration, and sprinting are regarded as critical technical skills and the main components of soccer training. The ability to sprint and to change direction while sprinting are determinants of performance in field sports, as evidenced by time and motion analysis (Sheppard and Young, 2006). In many sports, including soccer, athletes are required to accelerate, decelerate, and change direction throughout the game (Docherty et al., 1988). Often, these movements are performed in conjunction with passing, dribbling and striking movements (Abernethy and Russell, 1987; Farrow et al., 2005; Sheppard et al., 2006). Differences between higher and lower performers in anticipation and efficient decision making in accordance with sport-specific stimuli have also been mentioned in relevant literature (Abernethy and Russell, 1987; Tenenbaum et al., 1996; Farrow et al., 2005).

**Figure 1:**
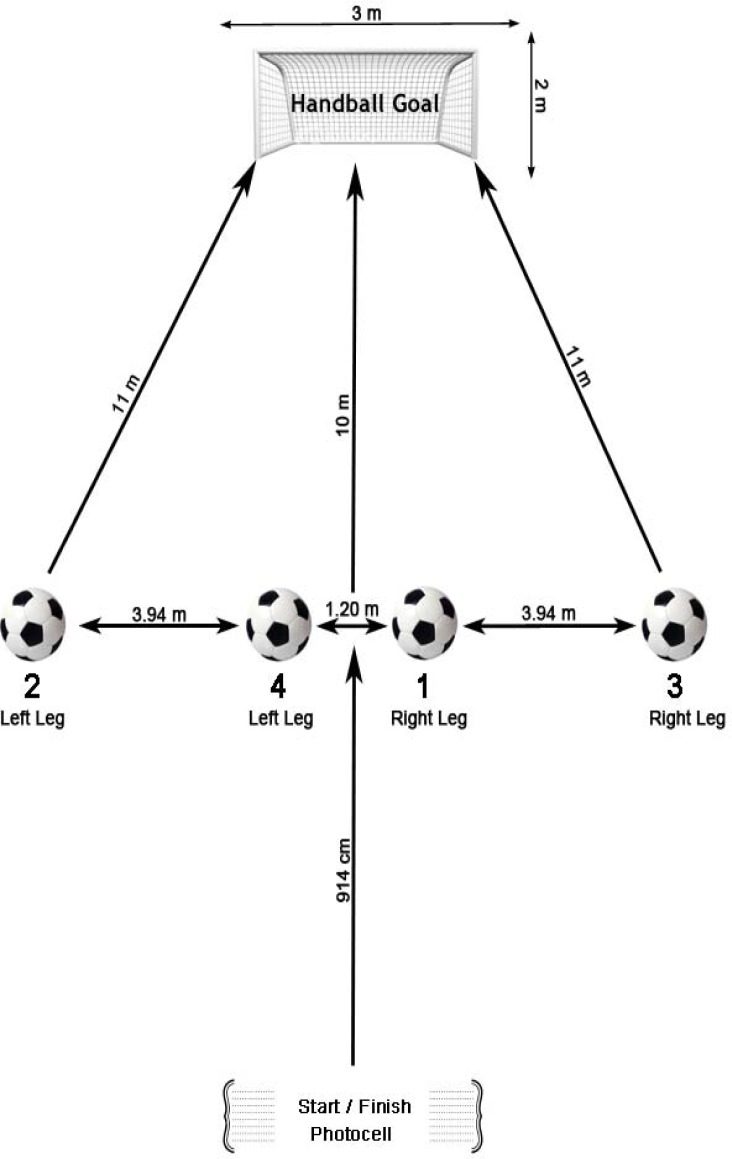
*A diagram and explanation of the new developed agility and skill test for soccer*.

In soccer agility, anticipating the direction and timing of the ball are crucial issues for success (Sheppard et al., 2006). However, few studies have evaluated sport-specific, physical performance tests of agility, including sprints, changes of direction and striking at the goal. Therefore, the purpose of this study was to develop and evaluate a novel test of agility and striking skill for soccer that involves sprint running, direction changing, and kicking stationary balls to the goal with accurate decision making. The classical T-drill agility test, developed by Semenick (1990), was implemented with four balls and the goal (Figure 1).

## Material and Methods

### Subjects

A total of 113 amateur (38) and professional (32) male soccer players from the Turkish League (Kirikkale-wide from Division 3 and 1^st^ Amateurs) (mean ± SD: age: 21.2 ± 3 years; body height: 1.78 ± 5.4 m; body mass: 72.2 ± 8.2 kg; body fat: 12.2 ± 3.9 %; years of experience: 6.8 ± 2.43) and university students (43) volunteered to participate in this study. The study protocol and methods were approved by the local institutional ethics committee of the University of Kirikkale, and all subjects gave written informed consent prior to participation.

### Testing Protocol

Kirikkale Soccer Club, with which the participating soccer players were affiliated, supported the study and provided detailed written approval. Subjects were also asked for written approval indicating their voluntary participation. All participants were tested during November 2010 as part of their athletic training program. All players became familiar with the testing procedures utilized in the current study before the official test was applied. The newly developed test and the other tests were practiced three times in the gym to ensure understanding and familiarization one week before the final test. All tests were performed on an indoor synthetic pitch. To prevent unnecessary fatigue, players were instructed to avoid intense exercise for 24 hours prior to the testing session. Body height, body mass and composition measurements were performed using the Tanita Body Composition Analyzer (Tanita Body Composition Analyzer BC 418 professional model, USA) from morning to noon on the test day. To assess the leg power of subjects, anaerobic work capacity was determined using the Wingate power test via a Monark 894E cycle ergometer (Monark, Stockholm) in the afternoon of the first testing day. On the second testing day, T-drill agility tests were conducted on all subjects twice. Furthermore, the athletes were exposed to the Urine Specific Gravity test with a new, pen-type refractometer to determine their hydration status (Atago, Tokyo, Japan). None of the participants was above the 1.025 gr/cm^3^ dehydrated value before evaluation. The main reason for choosing the T-drill classic test as a basis for a novel test was to determine agility outcomes and soccer striking skills. The T-drill classic test is widely utilized to measure speed with directional changes, such as forward sprinting, left- and right-side shuffling, and back pedaling. The Illinois agility test, which was also implemented in the current study, is commonly used to determine the ability to accelerate, decelerate, turn in different directions, and run at different angles. The Zigzag, Illinois, and T-drill classic tests were utilized because they require acceleration, deceleration, and the balance control aspects of agility. Additionally, these tests provide comparatively less of a learning effect (Miller et al., 2006). In addition to the rationales mentioned above, the reported validity and reproducibility of these tests suggests their suitability as comparison instruments in this study (Pauole et al., 2000; Roozen, 2004; Miller et al., 2006). Hence, considering the importance of agility and striking skill issues in soccer, a new test was required that was based on the T-drill agility test with the addition of balls and goals. A diagram of the newly developed agility and shooting skill test for soccer is shown in Figure 1. To prevent the sliding of balls and to ensure easy setting, a piece of rug was placed under the four balls. At the end of the test, success was determined based on the recorded time, as follows: if the subject could manage 4 goals with shooting legs, 1 second was subtracted from the recorded completion time for the newly developed T-drill Test; 0.75 seconds were subtracted for three goals, 0.50 seconds were subtracted for two goals and 0.25 seconds were subtracted for one goal. If the subject did not manage to strike the goal on any of four tries, the raw complete time score for the subject was recorded for the successful results of the T-drill with ball test.

Electronic timing gates were used to record the times (Tumer Electronic, Timing System, Ankara - Turkey). Subjects performed two trials of each test, with at least 2 minutes of rest between all trials and tests. A polar heart rate monitor (POLAR RS 400 MULTI Electro Oy, FIN-90440 KEMPELE, Finland) was used to control the rest interval. An interval heart rate of 110-125 was regarded as acceptable to initiate the test. This study was supported by the Institute of Scientific Research Projects at Kirikkale University.

### Statistical Analyses

Data analysis was performed using SPSS 16.0 for Windows (Chicago IL). Data obtained from the tests showed a normal distribution and were presented as mean ± standard deviation. A paired sample t-test was conducted to combine the results obtained from the test and re-test. The t-test was selected as the analytical method to determine speed with directional changes, such as forward sprinting, left- and right-side shuffling and back pedaling (13). A one-way analysis of variance (ANOVA) and Tukey post hoc tests were used to compare the different agility test results (classical T-drill, newly developed T-drill and shooting skill, and calculated success T-drill ball test). The relationship between performance on the agility test and the Wingate leg power test was analyzed by Pearson correlations (*r*). Coefficients of determination (*r^2^*) were used to interpret the meaningfulness of the relationships. A correlation coefficient (*r*) of 0.65 and above was considered high, an *r* of approximately 0.5 was considered moderate, while an *r* of 0.35 and below was considered low (9). The probability level for statistical significance was set at *p* ≤ 0.01.

## Results

Mean times (± SD) for all agility tests results are shown in Table 1. The results show that all tests revealed mean variances. The Illinois Test (16.28 s) revealed a high mean score for the agility tests, and the Zigzag test (6.09 s) showed a comparatively low mean value.

Table 2 shows the results of a variance analysis for five different agility tests and a sprint test among all participant groups. The results revealed a significant difference in terms of group means. An additional post hoc test (Scheffe) was applied to determine the within-group differences (Table 3). The Scheffe test results indicated that there were significant differences among all groups.

The correlation coefficient and coefficients of determination between the test-retest and among-test results of the participants are shown in Table 4. The correlation analysis results show no significant differences between the test and re-test results of various agility and sprint tests. A high level of reliability between the test and re-test results of the T-drill tests was observed (*r* = 0.97–0.99). However, low and moderate levels of correlations between various agility and sprint tests were observed (*r* = 0.26–0.54). Although a negative correlation was identified between T-drill success and anaerobic power tests (−0.12–0.18), there was no significant difference between them.

Furthermore, there were no significant differences between the left-leg (6/8 ± 1.3 goals, 75 %, *n* = 33 with left leg) and right-leg (7/8 ± 1.7 goals, 81 %, *n* = 80 with right leg) goal success in ball shooting (*p* > 0.05). Total ball shooting success to goal for both legs was 6.2 / 8 ± 1.3 goals, 78 %, *n* = 113.

The mean scores of the agility tests are shown in Table 5. An ANOVA test revealed no significant differences between the results of the agility tests (*p* < 0.01). Based on the agility tests (classic T-drill, T-drill with ball, T-drill ball Success, Zigzag, and Illinois), significant differences were observed among the studied groups (professionals, amateurs, and students).

## Discussion

This study aimed to develop and evaluate a new test of agility and shooting skill for soccer that involved sprint running, direction changing and kicking a stationary ball to a goal with accurate decision making. Although agility performances on the classic T-drill, T-drill ball, T-drill success, Illinois, and Zigzag tests were all correlated at statistically significant levels (*p* < 0.01) (Table 4), the level of correlation cannot be regarded as high. That is to say, the correlation among agility tests is within the low to moderate levels (Kowacks et al., 1999). Similar to relevant literature, remarkably high test-retest values for the agility tests were obtained. For example, correlation values around 0.94 were observed for the T-drill ball test, which is higher than the results of other studies that measured the reliability of tests involving unplanned direction changes (Chelladurai et al., 1977; Docherty et al., 1988; Hertel et al., 1999; Pauole et al., 2000; Farrow et al., 2005). All agility and sprint tests produced correlation values that were acceptably reliable (*p* > 0.85) for physical performance tests (Thomas and Nelson, 2001). The significant difference between test 1 and test 2 results and the high test-retest correlation value (*r* = 0.94) indicates that the newly developed T-drill test provides acceptable test-retest reliability and inter-rater reliability. Similarly, data for the agility tests and sprint tests suggest that different types of agility and sprinting tests have common traits in terms of their physiological and biomechanical determinants. However, the coefficients of determination indicate that the classic T-drill test and the T-drill ball tests share only 18-23 % common variance. In other words, despite the fact that a significant correlation was obtained between these two tests, the overall variance in the correlation is just one-fourth of the common variance (***r****^2^*). The results also suggest that there is no significant correlation between the agility and power tests, which is supported by literature (Little and Williams, 2005). Thomas and Nelson (2001) stated that when the common variance between two variables is less than 50 %, it means that they are specific or somewhat independent in nature. Another study focusing on agility, conducted by Little and Williams (2005), obtained a slightly faster total completion time compared to the current study. This may be because the participants in that study were soccer players from the first and second division English League. It appears that the T-drill with ball test and the peak power Wingate test show relatively independent attributes for amateur and professional soccer players. In contrast, the Illinois test results show that participants performed above average (Davis et al., 2000).

Analysis of results also indicates that the differences between the durations of the T-drill classic (9.84 ± 0.41), the T-drill ball (12.11 ± 0.55), and the T-drill success (11.34 ± 0.63) suggest that the newly developed T-drill test requires additional skills of ball striking to target and decision making. Thus, the new test produces less successful performance in recorded time results compared to the T-drill classic test (*p* < 0.01). The differences in scores between the T-drill tests with and without balls can be explained by the differences in decision making and the skill of kicking the ball. The athletes are required to make decisions on correct shooting to goal with both legs related to their physical and cognitive abilities. In other words, many athletes seemed to complete the decision-making process at an earlier time during the test (Tenenbaum et al., 1996; Sheppard and Young, 2006; Jordet et al., 2007). The low common variance between the T-drill classic and T-drill ball tests supports the validity of the newly developed agility and skill test. Although all of the agility tests were designed to measure quickness and agility, the newly developed test measured not only agility, acceleration and directional changes of the subjects but also quick and accurate striking skills.

## Practical Applications

Assessing changes in direction during sprints and shuttle runs by the sprint, Zigzag, Illinois, and T-drill classic tests are unlikely to be adequate for determining the physical and cognitive skills required in soccer. Thus, a test involving agility and ball-striking skill with sport-specific characteristics is missing. The current study aimed to respond this lack by developing a new test to assess on-field skill and agility performance. The new test, compared to other agility tests, involves some further cognitive and physical skills such as striking the ball with a quick, well-timed and accurate decision. Soccer coaches may prefer the newly developed T-drill agility and skill test because it allows them to assess their players in terms of quick and proper decision making and to provide further training solutions for low-level soccer players. This test may also be used for talent identification in youth soccer players.

## Figures and Tables

**Table 1:** Descriptive statistics for agility and sprint tests’ scores

	N	Mean	Std. Deviation	Minimum	Maximum
T-drill Classic (Sc)	113	9.84	.41	8.55	10.53
T-drill with Ball (Sc)	113	12.11	.55	10.45	13.20
T-drill Ball Success (Sc)	113	11.34	.63	9.45	12.55
Zigzag Test (Sc)	113	6.09	.49	5.14	7.46
Ilionis Test (Sc)	113	16.28	.57	15.34	17.54
30 m (s)	113	4.23	.57	3.16	5.56

**Table 2:** Variance Analysis for five different agility tests among all groups

	Sum of Squares	df	Mean Square	F	Sig.
Between Groups	6175.023	4	1543.756	5303.044	.000
Within Groups	163.020	560	.291		
Total	6338.043	564			

Significance level (p< 0.01)

**Table 3:** Post hoc results for five different agility tests

Dependent Variable: All agility tests				

	(I) Group	(J) Group	Mean Difference (I–J)	Sig.
Scheffe	(1) T-drill Classic	2.00	−2.27540^*^	.000
		3.00	−1.50133^*^	.000
		4.00	3.74770^*^	.000
		5.00	−6.44637^*^	.000
	(2) T-drill with Ball	1.00	2.27540^*^	.000
		3.00	.77407^*^	.000
		4.00	6.02310^*^	.000
		5.00	−4.17097^*^	.000
	(3) T-drill Ball Success	1.00	1.50133^*^	.000
		2.00	−.77407^*^	.000
		4.00	5.24903^*^	.000
		5.00	−4.94504^*^	.000
	(4) Zigzag Test	1.00	−3.74770^*^	.000
		2.00	−6.02310^*^	.000
		3.00	−5.24903^*^	.000
		5.00	−10.19407^*^	.000
	(5) Ilionis Test	1.00	6.44637^*^	.000
		2.00	4.17097^*^	.000
		3.00	4.94504^*^	.000
		4.00	10.19407^*^	.000

Significance level (p< 0.01)

**Table 4:** The relationships between tests-re-test and agility test of soccer players

**Relationship assessed**		**r**	**r^2^**	**p value**
T-drill Classic	T-drill with Ball (s)	0.42^**^	0.18	P<0.000
T-drill Classic	T-drill success (s)	0.48^**^	0.23	P<0.000
T-drill ball	T-drill Success (s)	0.91^**^	0.83	P<0.000
T-drill Success	Zigzag (s)	0.54^**^	0.29	P<0.000
T-drill Success	Illinois (s)	0.26^**^	0.07	P<0.006
T-drill Success	30 m Sprint (s)	0.48^**^	0.23	P<0.000
T-drill Success	Peak Anaerobic Power (W/kg)	−0.18	0.04	P>0.071
T-drill Success	Avg. Power (W/kg)	−0.12	0.01	P>0.193
T-drill Classic (s) 1	T-drill Classic (s)2	0.97^**^	0.94	P<0.000
T-drill ball (s) 1	T-drill ball (s) 2	0.94^**^	0.88	P<0.000
T-drill Success 1	T-drill Success 2	0.89^**^	0.79	P<0.000
Zig zag (s) 1	Zig zag (s) 2	0.98^**^	0.96	P<0.000
Illinois (s) 1	Illinois (s) 2	0.98^**^	0.96	P<0.000
30 m (s) 1	30 m (s) 2	0.91^**^	082	P<0.000

Significant correlation in (p< 0.01) level.

**Table 5:** Variance Analysis among various subjects groups

	Professional N: 32	Amateurs N: 38	Students N: 43	P
T-drill (Classic)	9.48±0.38	9.98±0.30	10.00±0.33	P<0.01^*^
T-drill with Ball	11.74±0.61	12.20±0.55	12.36±0.33	P<0.01^*^
T-drill Ball Success	10.90±0.65	11.36±0.66	11.70±0.34	P<0.01^*^
Zigzag Test	5.60±0.31	6.11±0.32	6.45±0.42	P<0.01^*^
Illinois Test	16.01±0.62	16.30±0.57	16.54±0.41	P<0.01^*^

* Significant differences among two soccer groups and a control group (ANOVA)

* Significance level (p<0.01)
